# An Efficient Signature Based on Necroptosis-Related Genes for Prognosis of Patients With Pancreatic Cancer

**DOI:** 10.3389/fgene.2022.848747

**Published:** 2022-03-28

**Authors:** Heng Shi, Qin Peng, Xianling Zhou, Yushan He, Shengyun Sun

**Affiliations:** ^1^ Department of Traditional Chinese Medicine, The First Affiliated Hospital of Jinan University, Jinan University, Guangzhou, China; ^2^ Department of Gastroenterology, The Central Hospital of Shaoyang, University of South China, Shaoyang, China

**Keywords:** bioinformatics, necroptosis, pancreatic cancer, prognosis, risk model

## Abstract

Pancreatic cancer (PCa) is a highly lethal and aggressive disease, characterized by high mortality rates. Although necroptosis plays a vital role in tumor progression, cancer metastasis, prognosis of cancer patients, necroptosis-related gene (NRG) sets have rarely been analyzed in PCa. Therefore, definition of novel necroptosis-related prognostic markers for PCa patients is urgently needed. Here, we screened 159 NRGs and identified 132 differentially expressed NRGs in The Cancer Genome Atlas (TCGA) and Genotype-Tissue Expression (GTEx) cohorts. Next, we employed univariate and multivariate Cox proportional regression models to establish a prognostic-related NRG signature comprising five NRGs that could stratify patients into high-risk and low-risk groups. Results from survival analysis showed that patients in the high-risk had dramatically shorter overall survival (OS) rates compared with their low-risk counterparts. Results from univariate and multivariate Cox regression analysis further confirmed the independent prognostic value of the established necroptosis-related signature, and the area under receiver (AUC) of the operating curve (ROC) for 1-, 3-, 5-years was 0.72, 0.74, and 0.75, respectively. Finally, we validated the signature efficacy using an independent cohort from the Gene Expression Omnibus (GEO) database. The ROC curve confirmed the predictive capacity of the five-gene signature. Furthermore, we validated expression of the signature proteins using the Human Protein Atlas (HPA) database. In conclusion, we successfully constructed a novel necroptosis-related signature for prognosis of patients with pancreatic cancer.

## Introduction

Pancreatic cancer (PCa) is a malignant tumor of the digestive system characterized by a low prevalence, a high degree of malignancy, rapid disease progression, poor prognosis, and high fatality rate (Vincent et al., 2011; Yadav and Lowenfels, 2013). Treatment options for PCa patients are limited, with surgical resection the only potential cure (Hartwig et al., 2013). To date, however, many PCa patients miss the opportunity for surgery owing to difficulty in early diagnosis, and combining traditional chemotherapies with new therapies that directly targeted against the molecular changes in PCa seems to be the most promising treatment now (Kleespies et al., 2006; Strimpakos et al., 2008). Therefore, development of an effective prediction model is imperative to assess patients’ prognosis with PCa. In this way, effective treatment can be chosen to balance side effects and survival rates and to determine whether more aggressive treatment should be implemented. Advances in oncogenomics have led to the development of new prognostic related genes that reflect progression at the molecular level and may contribute to more accurate predictions of individual survival.

Previous studies have shown that apoptosis is a programmed cell death mechanism, a natural barrier that prevents the development of cancer (Hanahan and Weinberg, 2011). Unlike apoptosis, necrosis is uncontrollable and a way of death defined by morphological characteristics. Necroptosis, which is regulated necrosis, was first discovered and named by Degterev et al. (2005) represents a new concept of cell death. Notably, necroptosis is mechanically similar to apoptosis and morphologically similar to necrosis (Christofferson and Yuan, 2010), and has been shown to play an important role in regulating cancer biology, including oncogenesis, tumor metastasis, tumor immunity, and tumor subtypes (Seehawer et al., 2018). Previous studies have shown that, as a combination of apoptosis and necrosis, the key mediators of the necroptosis pathway alone or in combination promote both tumor metastasis and progression (Strilic et al., 2016). However, necroptosis has also been shown to offer protection against tumor development when apoptosis is compromised (Höckendorf et al., 2016). Specifically, necroptosis has dual effects on cancer. Considering its important role in tumor biology, necroptosis has become a new target for tumor treatment. Moreover, based on current reports, more and more compounds and a variety of therapeutic drugs can prevent tumor by inducing or manipulating necroptosis (Fulda, 2014).

In the present study, we comprehensively analyzed necroptosis-related genes (NRGs) in PCa, and identified five genes related to patients’ prognosis. We used these NRGs to establish a prognostic signature and found that it was able to divide PCa patients into two groups with significant molecular and prognostic differences.

## Materials and Methods

### Human Necroptosis-Related Gene Set

We searched the Kyoto Encyclopedia of Genes and Genomes (KEGG) database (https://www.kegg.jp/) and identified 159 genes in the necroptosis pathway. A summary of these genes is provided in Supplementary Table S1.

### Data Source

The Cancer Genome Atlas (TCGA) data of pancreatic cancer and Genotype-Tissue Expression (GTEx) data of healthy donators were downloaded from UCSC Xena database (http://xena.ucsc.edu/). The transcriptome data from the TCGA database were normalized with the log_2_ (x+1) transformation. GSE71729 dataset was downloaded from Gene Expression Omnibus (GEO) (http://www.ncbi.nlm.nih.gov/geo) after systematically screening for validation of the established signature. The criteria were as follows: the information of samples, including survival state and survival time, was relatively complete and substantial; the samples of PCa patients were over 100.

### Data Preprocessing

Raw data from TCGA and GTEx were preprocessed using the following criteria: (1) genes were excluded if the Fragments per Kilobase Million (FPKM) value was zero in more than half of the samples; (2) genes with missing expression values in over 30% of samples were removed; (3) samples without related clinical data or OS < 30 days were excluded; and (4) normal tissue samples from TCGA dataset were removed. The GEO dataset was used as an external validation cohort.

### Identification of Differentially Expressed Genes

Differentially expressed genes in TCGA and GTEx datasets were calculated using the “*limma”* R package (Ritchie et al., 2015), while DEGs in GEO dataset were identified using the “*GE O 2R*.” DEGs with absolute log2 fold change (FC) > 1 and adjusted *p* value <0.05 were included for subsequent analyses.

### Functional Enrichment Analysis

Gene Ontology (GO) enrichment, Kyoto Encyclopedia of Genes and Genomes (KEGG) pathway, and Gene Set Enrichment Analysis (GSEA) analyses were performed for identification of potential biological processes (BP), cellular components (CC), molecular functions (MF), and signaling pathways regulated by the differentially expressed NRGs. These analyses were performed using “*clusterProfiler”* R package (Yu et al., 2012), while the enrichment terms were visualized using the ‘*GOplot’* R package (Walter et al., 2015).

### Construction of an NRG Related Prognostic Model

We employed a univariate Cox proportional hazard regression model to assess the relationship between overall survival (OS) and expression levels of differentially expressed NRGs. Next, we employed a multivariate Cox proportional hazards regression model using the candidate prognostic NRGs identified by univariate regression analysis to obtain independent prognostic factors and calculate regression coefficient as well as hazard ratios (HRs).

We constructed prognosis-related NRGs using multivariate cox regression. Next, we incorporated the expression values for each gene and developed the following prognostic formula for PCa, based on the identified NRGs and their corresponding coefficients: risk score = ∑ (coefficient_i_  ×  expression of signature gene_i_). We used the median risk score as the cut-off point to stratify PCa patients into low-risk and high-risk groups. Then, we applied the Kaplan-Meier method to assess and compared survival differences between the two groups using log-rank statistical. Moreover, we applied the multivariate cox regression model and stratified analysis to determine the role of risk scores in predicting patients’ prognosis, with ROC curves generated to determine the predictive accuracy of the models using “*timeRO*” R package.

### Statistical Analysis

Survival curves were generated by Kaplan-Meier method and compared by log-rank test. Cox proportional hazard model was used for multivariate analysis. All statistical analyzes were performed using the R software (Rx64 4.0.3, https://www.rstudio.com/) with statistical significance set at *p* < 0.05.

## Results

### Differentially Expressed NRGs

**FIGURE 1:**
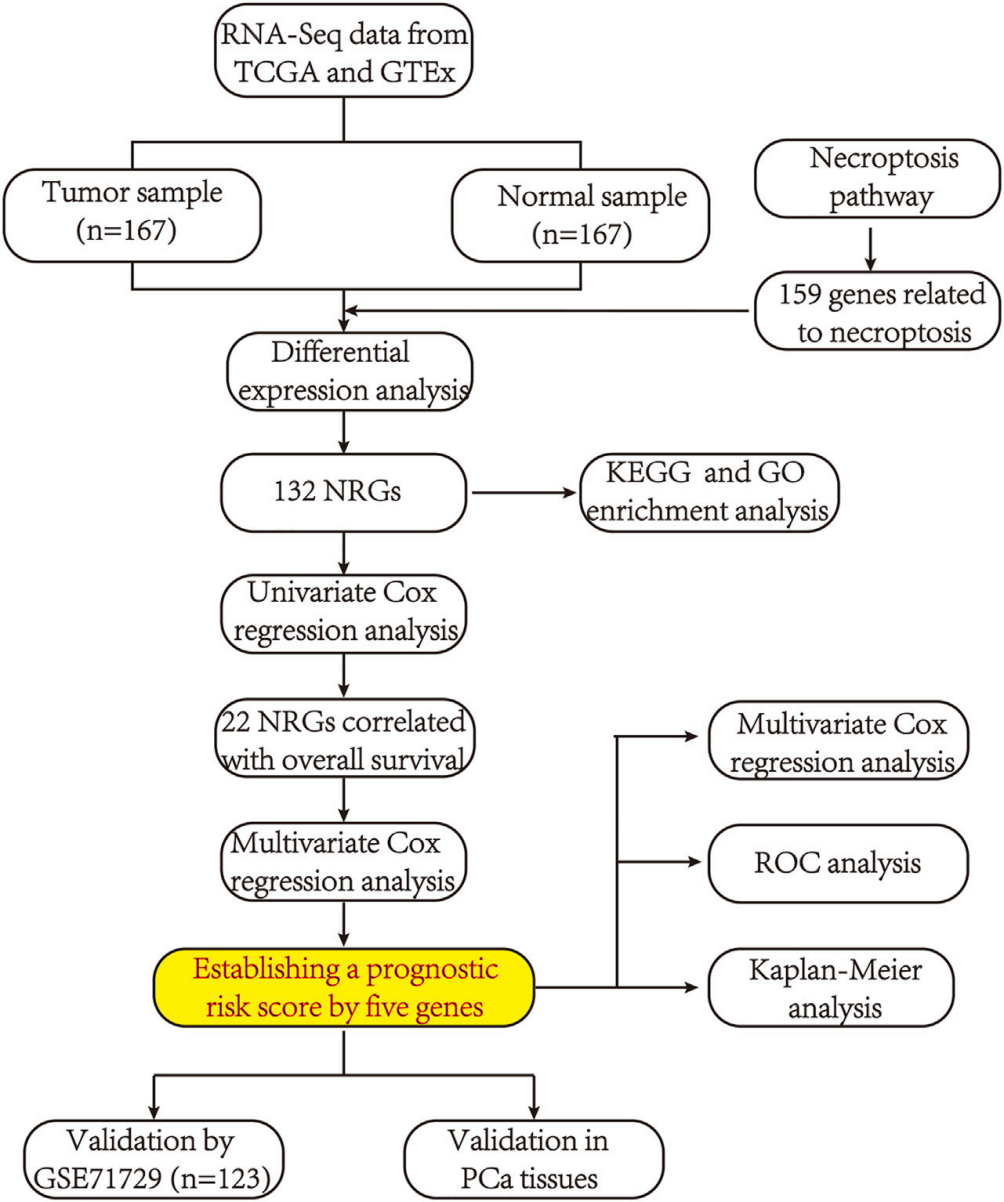
A flowchart showing the procedure of this study.

**FIGURE 2:**
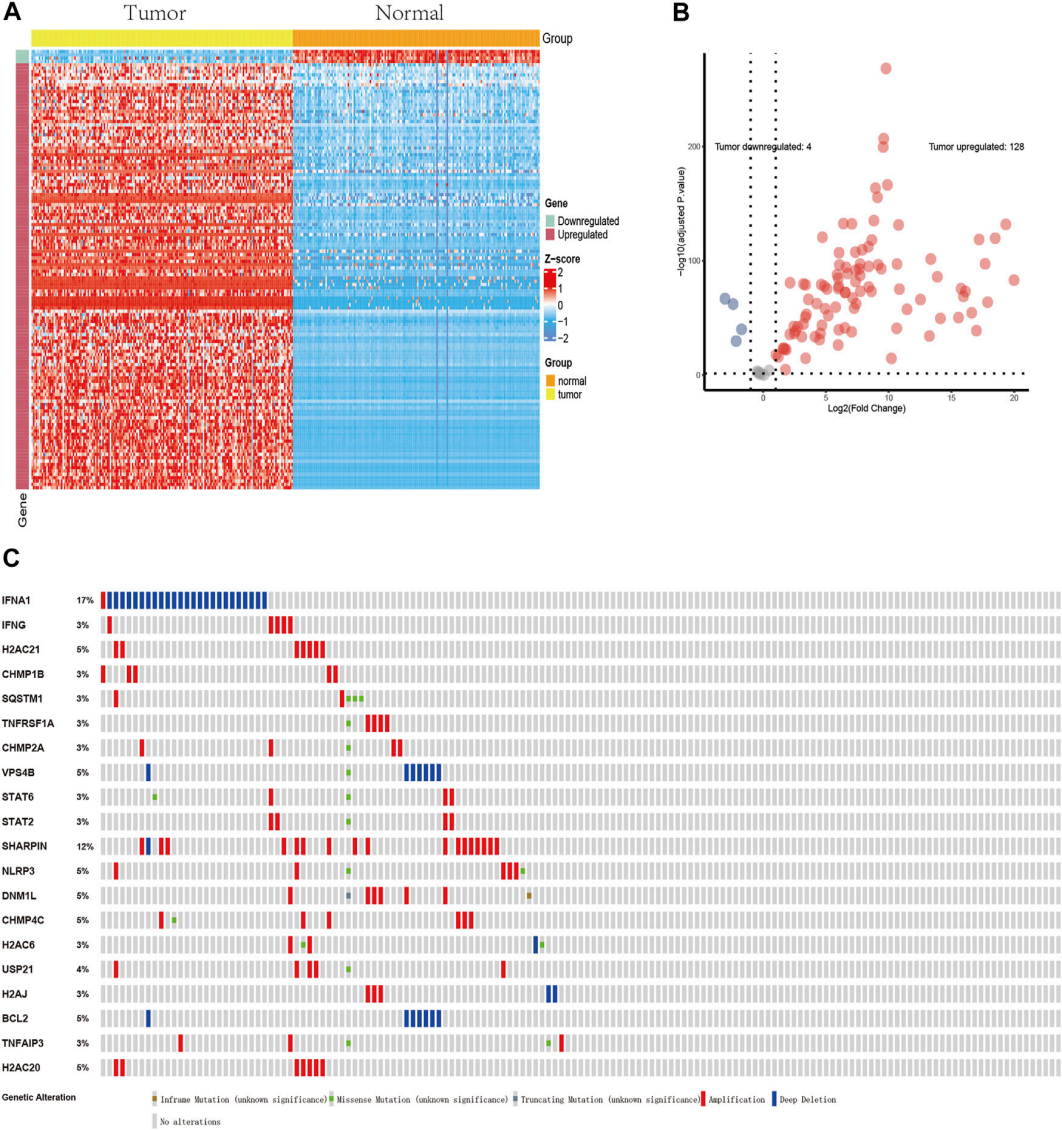
Profiles of differentially expressed NRGs. **(A)** A heatmap of 132 differentially expressed NRGs. **(B)** A volcano plot showing the differentially expressed NRGs. 128 red dots indicate upregulated NRGs and four blue dots indicate downregulated NRGs in PCa compared with normal tissues. **(C)** Profiles of mutations in NRGs. A total of 20 genes exhibited a mutation rate ≥3%.

The flow chart of the present study is shown in Figure 1. After screening and classification, RNA-seq data and clinical information of 167 pancreatic cancer samples and 167 normal samples were included for subsequent analyzes. Clinical characteristics of the included patients are presented in Table 1. We extracted expression values of 159 NRGs from PCa patients, of which 128 were upregulated NRGs and four were downregulated NRGs Compared with normal samples (Figure 2A, Supplementary Table S2). Volcano plot revealed the expression patterns of these differentially expressed NRGs between tumor and normal tissues (Figure 2B). Owing to the important clinical information of these NRGs, we used cBioPortal database (https://www.cbioportal.org/) to search for genetic alterations of these genes, and found that amplification and deep deletion were the two most common types of mutations. Notably, a total of 20 genes had a mutation rate greater than 3% (Figure 2C), with IFNA1 being the most frequently mutated gene (17%).

**TABLE 1:** Patient characteristics in the TCGA-PAAD cohorts.

Characteristics	—	Number of Cases
Age (years)	≥60	113
	<60	54
Gender	Female	76
	Male	91
Tumor T	T1	6
	T2	21
	T3	137
	T4	3
Tumor N	N0	46
	N1	118
	Nx	3
Tumor M	M0	77
	M1	4
	Mx	86
Tumor stage	Stage i	18
	Stage ii	142
	Stage iii	3
	Stage iv	4
Survival status	Alive	76
	Dead	91

### Functional Enrichment of the Differentially Expressed NRGs

**FIGURE 3:**
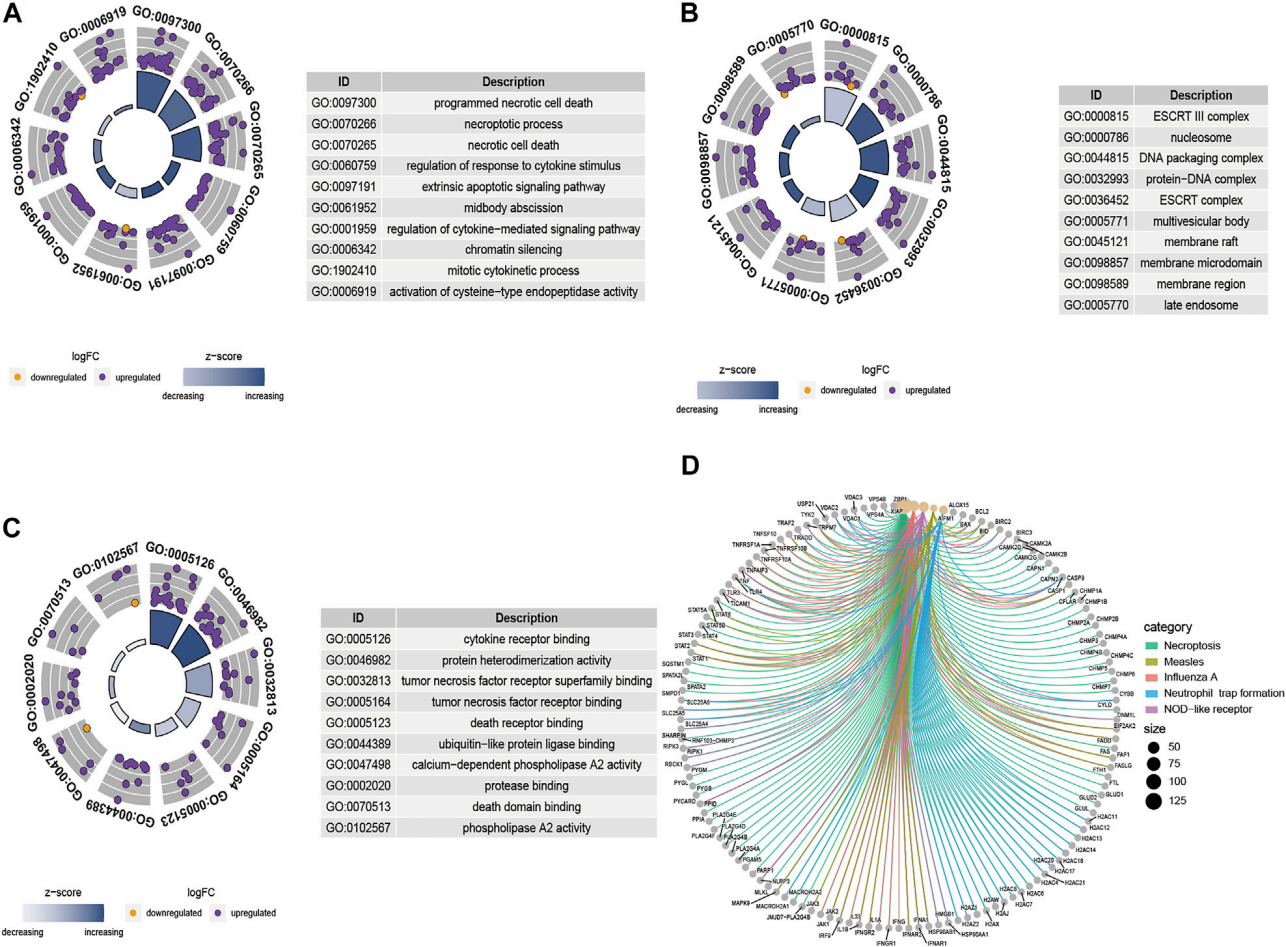
GO term and KEGG pathway analysis results based on the 132 differentially expressed NRGs. **(A)** The GO circle shows the scatter map of the logFC of the specified genes in biological process (BP), **(B)** cellular component (CC), and **(C)** molecular function (MF). A higher the Z-score value implies higher expression of the enriched pathway, and purple and orange dots represent upregulated and downregulated NRGs, respectively. **(D)** The KEGG circle revealed the most significant pathway correlated with differentially expressed NRGs. Lines of different colors represent different signal pathways, and around the dot are annotations for the names of genes.

Results of GO functional enrichment and the KEGG pathway analysis of these differentially expressed NRGs are shown in Figure 3. Summarily, the top enriched GO terms in biological processes are programmed necrotic cell death, necroptotic process, and necrotic cell death (Figure 3A). In terms of cellular components, genes were mostly enriched in ESCRT III complex and nucleosome (Figure 3B). Cytokine receptor binding and protein heterodimerization activity were the most enriched molecular function (Figure 3C). Results from KEGG pathway analysis revealed these genes were notably associated with pathways related to necroptosis, measles, influenza A, neutrophil trap formation, and NOD-like receptor (Figure 3D).

### Establishment of a Necroptosis-Related Prognostic Signature in PCa

**FIGURE 4:**
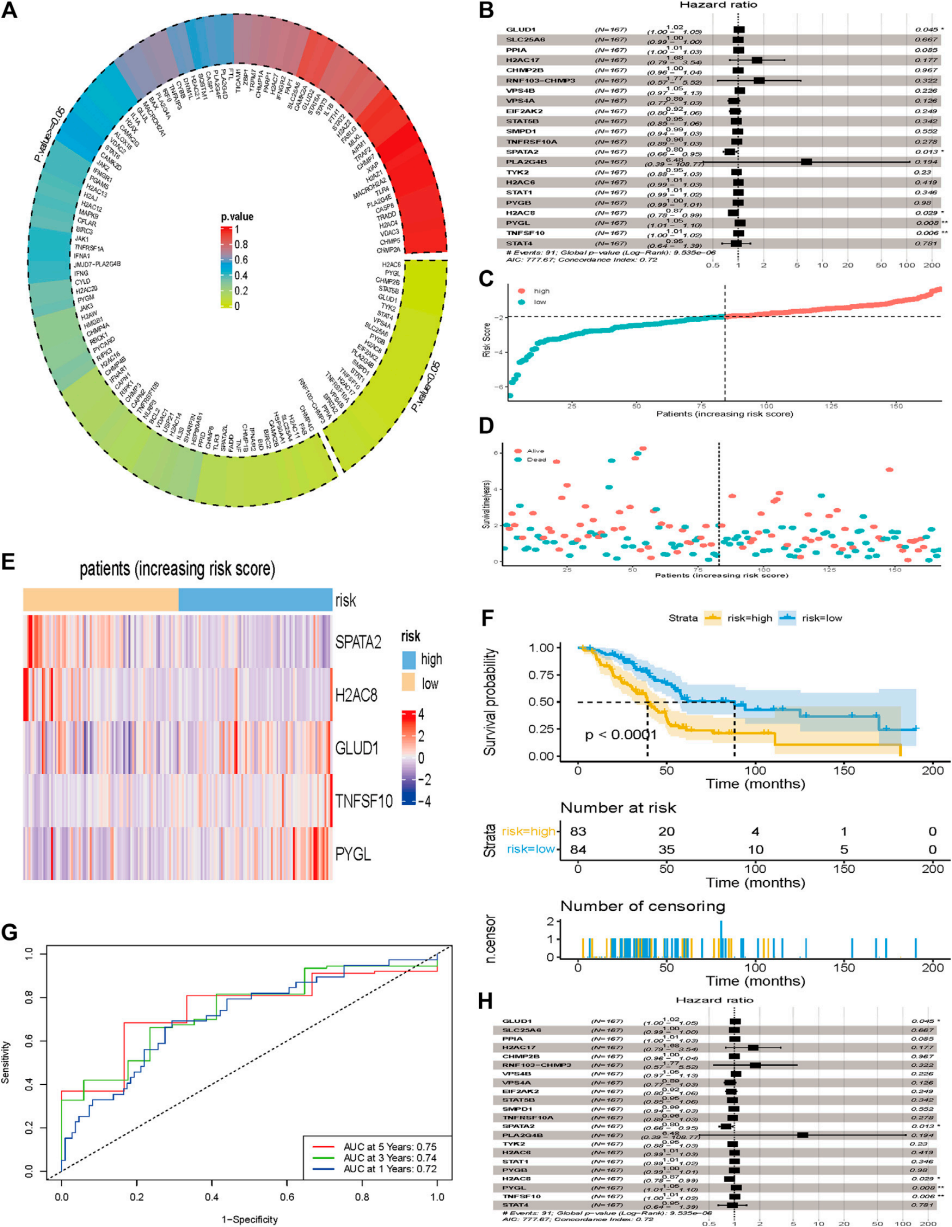
Development of a prognostic index based on the identified NRGs. **(A)** 132 differentially expressed NRGs were included in the univariate Cox regression analysis and results showed that 22 NRGs at the bottom right of the plot circled by dotted line were correlated with overall survival (*p* < 0.05). **(B)** 22 NRGs screened by univariate Cox regression analysis were included in the multivariate Cox regression analysis and results showed that 5 NGRs (GLUD1, SPATA2, H2AC8, PYGL, and TNFSF10) were independent factors for prognosis of PCa. **(C)** The number of patients in different risk groups. **(D)** Survival status of patients across different groups. **(E)** Heatmap showing the expression profile of the 5 DRGs. **(F)** Kaplan-Meier curves of overall survival of patients in the high- and low-risk groups, stratified by the necroptosis-related signature in the cohorts. **(G)** A forest plot of multivariate Cox regression analysis in the cohorts**. (H)** ROC curves of overall survival for the NRG signature. The AUC values for 1-, 3- and 5- years in the TCGA cohorts were 0.72, 0.74 and 0.75, respectively.

Results of the univariate Cox regression analysis, performed to evaluate these differentially expressed NRGs and identify key genes associated with the prognosis of PCa, are shown in Figure 4A. A total of 22 prognostic NRGs, namely H2C6, PYGL, CHMP2B, STAT5B, GLUD1, TYK2, STAT4, VPS4A, SLC25A6, PYGB, H2AC8, EIF2AK2, PLA2G4B, SMPD1, STAT1, TNFSF10, H2AC17, TNFRSF10A, VPS4B, SPATA2, PPIA, and RNF103-CHMP3, were statistically significant based on univariate Cox regression analysis, thus were further included in the subsequent multivariate Cox regression analysis. Consequently, results of multivariate Cox regression analysis revealed a total of five genes, namely GLUD1, SPATA2, H2AC8, PYGL, and TNFSF10, were significantly associated with prognosis (Figure 4B, Supplementary Table S3). According to the multivariate Cox proportional hazards regression model, our prognostic model for predicting prognosis based on the five genes was formed using the following formula: risk score = (0.0243 × expression level of GLUD1) + (−0.2283 × expression level of SPATA2) + (−0.1336 × expression level of H2AC8) + (0.0533 × expression level of PYGL) + (0.0104 × expression level of TNFSF10).

Next, we calculated the risk scores for each patient, and used the median risk value as a cut-off point to stratify patients into either the low-risk (*n* = 84) or high-risk groups (*n* = 83). The expression values of the five NRGs and the distribution of risk scores are presented in Figures 4C,D. Result showed that patients in the high-risk group were associated with higher death rates compared with patients in the low-risk group. Figure 4E showed the expression profile of the five NRGs between low- and high-risk group. Kaplan-Meier survival curves showed that patients in the low-risk group were associated with longer OS than their high-risk counterparts (Figure 4F). The AUC values for 1-, 3- and 5- years in the TCGA cohorts were 0.72, 0.74 and 0.75, respectively (Figure 4G).

### Prognostic Value of the Established NRG-Related Signature

**FIGURE 5:**
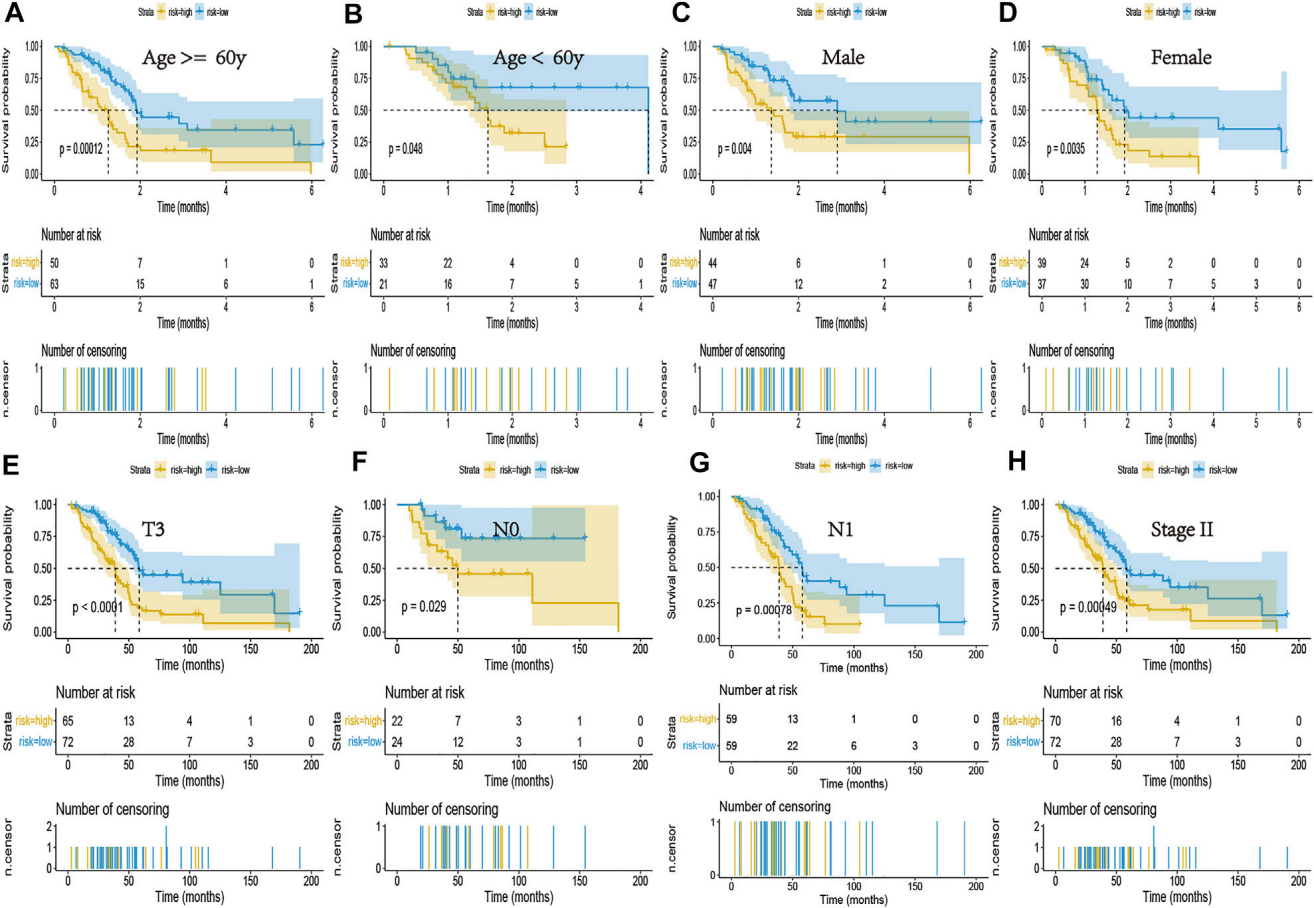
Kaplan-Meier curves of the NRG signature in patients stratified by age, gender, and tumor stage. **(A,B)** Differences in OS between patients in the high- and low-risk groups, stratified by age. **(C,D)** Differences in OS between patients in the high- and low-risk groups, stratified by gender. **(E)** Difference in OS between patients in the high- and low-risk groups, stratified by T3 stage. **(F,G)** Difference in OS between patients in the high- and low-risk groups, stratified by N stage. **(H)** Difference in OS between patients in the high- and low-risk groups, stratified by Stage II.

We incorporated the risk scores, age, gender, and tumor stage into the multivariate Cox model and verified that the NRGs signature was a significant and independent factor for prognosis of patients with PCa (*p* < 0.001) (Figure 4H). Based on clinical characters, including age, gender and tumor stage, we stratified the whole cohort from TCGA to determine the applicability of this signature. Stratification analysis, based on age, gender, and tumor stage, showed patients in high-risk group had significantly shorter OS than that in low-risk group patients (Figures 5A–H). The number of T1, T2, T4, stage I, and stage III-IV patients was too small, and almost all the pancreatic cancer were ductal adenocarcinoma, thus we did not include them in further analysis. Taken together, the established necroptosis-related signature was feasible and reliable in predicting survival in multiple strata of patients.

### Validation of the Necroptosis-Related Signature Using an Independent Cohort

**FIGURE 6:**
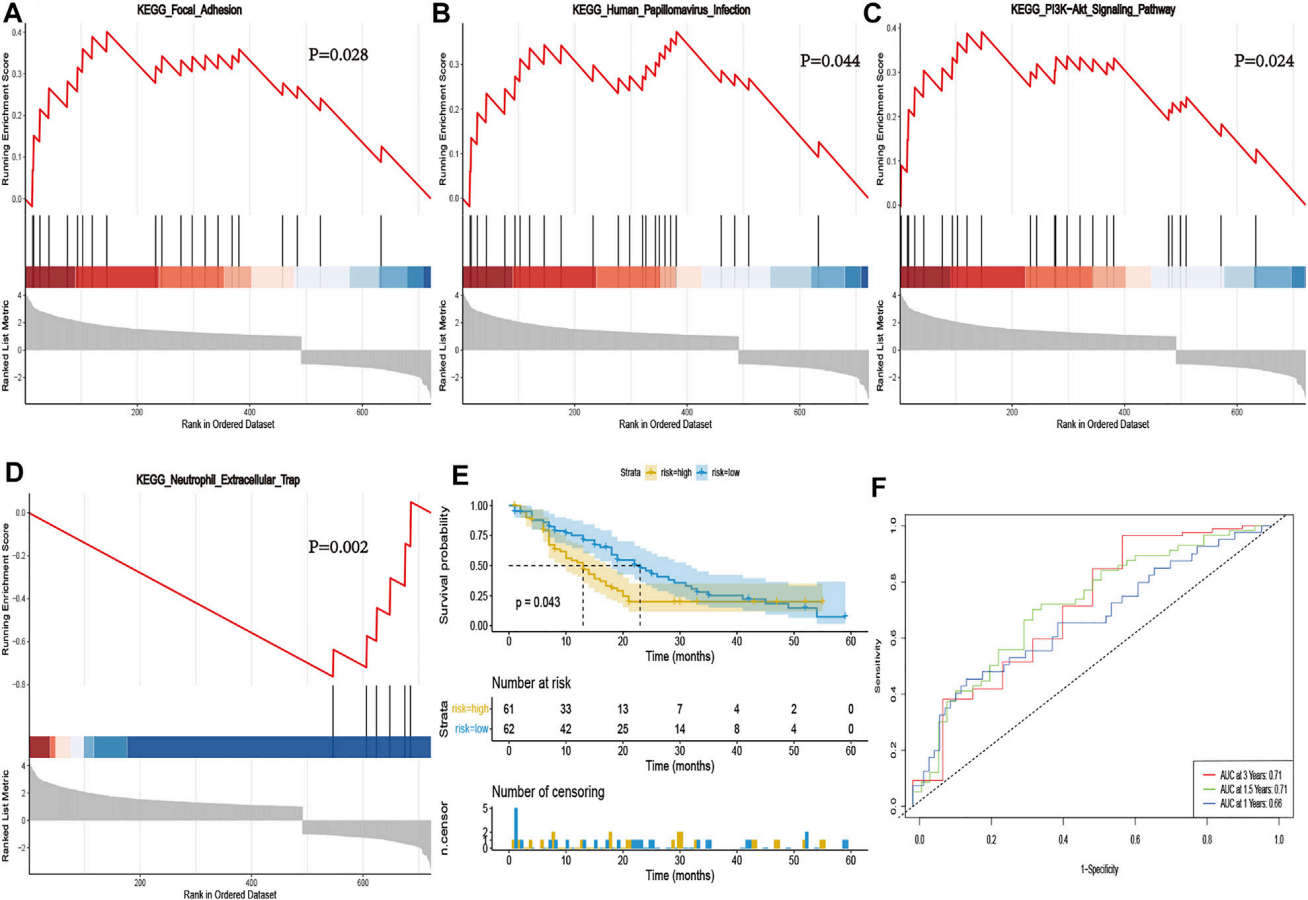
Validation of the necroptosis-related signature in GSE71729. GSEA validated activity of focal adhesion **(A),** human papillomavirus infection **(B),** PI3K-Akt signaling pathway **(C)**, and neutrophil extracellular trap **(D)**. **(E)** Kaplan-Meier curve of OS in the high- and low-risk groups stratified by the necroptosis-related signature in GSE71729. **(F)** The ROC analysis in GSE71729. The AUC values for 1-, 1.5- and 3- years were 0.66, 0.71 and 0.71 in the GSE71729 cohorts, respectively.

We performed GSEA analysis to identify necroptosis associated pathways, based on the differentially expressed genes between PCa and normal tissues in the GSE71729 cohort. Results revealed several significantly enriched necroptosis-related pathways, included focal adhesion, human papillomavirus infection, PI3K-Akt signaling pathway, and neutrophil extracellular trap (Figures 6A–D). Thereafter, we calculated risk scores for each patient in the GSE71729 dataset, using the same formula, and used the median risk score to stratify the patients into either a high- or low-risk groups based on. Kaplan-Meier curves confirmed the prognostic ability of our signature (Figure 6E). However, we did not perform multivariate Cox analysis of other clinical factors, due to a lack of clinical phenotype. The AUC values for 1-, 1.5- and 3- years were 0.66, 0.71 and 0.71 in the GEO cohorts, respectively (Figure 6F). Collectively, these results validated that our risk signature had excellent prognostic ability in PCa patients.

### Validation in PCa Tissues

**FIGURE 7:**
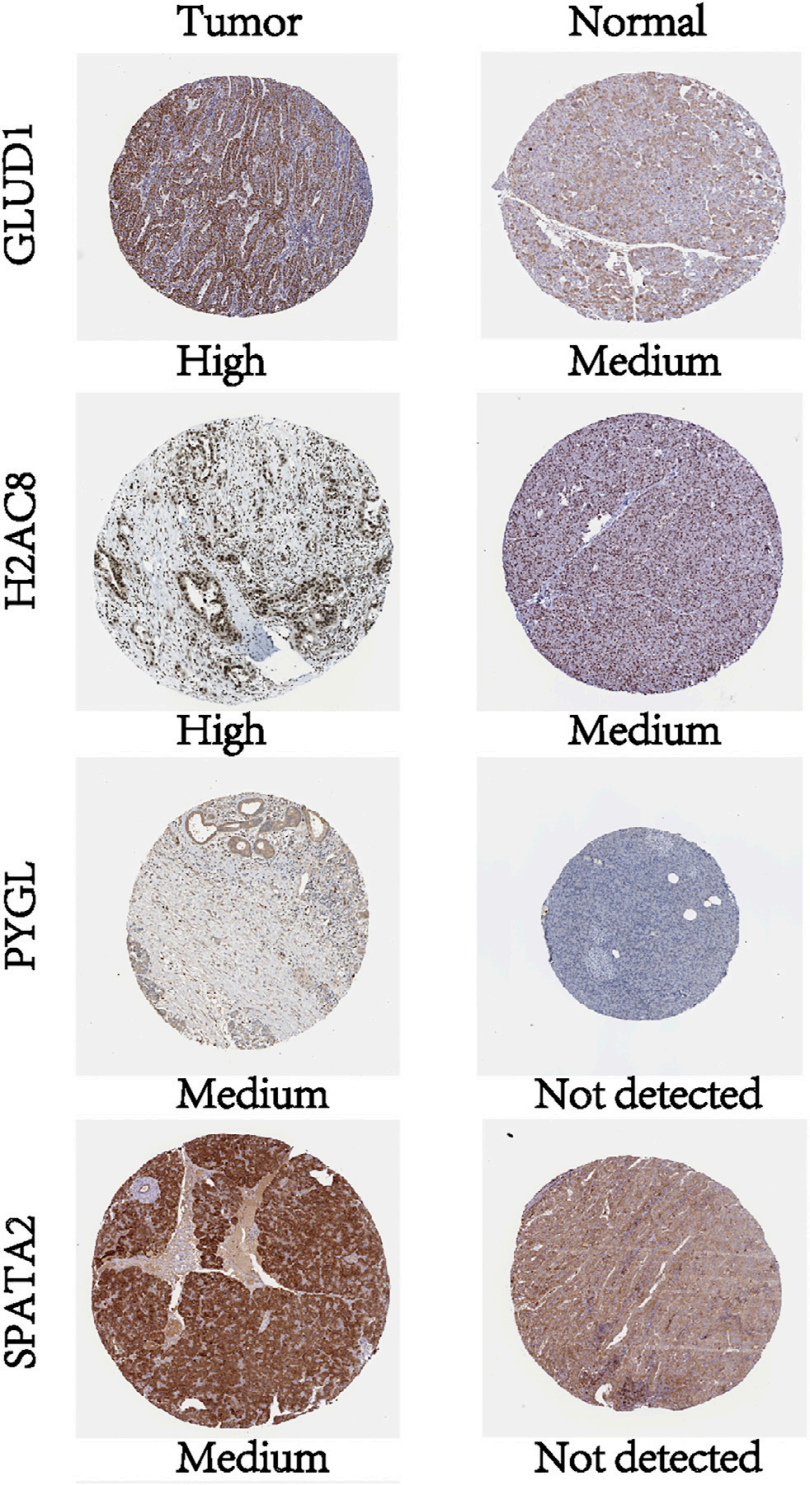
Profiles of protein expression of the four NRGs in PCa and normal tissues based on immunohistochemistry results from the Human Protein Atlas.

To investigate the protein expression of key NRGs, we studied the immunohistochemistry results using the Human Protein Atlas database (https://www.proteinatlas.org/) in tumor and normal pancreatic tissues. Results revealed that all other proteins, except TNFSF10, were expressed with profiles consistent with the results from the TCGA database and GTEx database (Figure 7).

## Discussion

Numerous studies have reported various modes of cell death among organisms, of which apoptosis is a common occurrence. Another mode, known as necroptosis, is a necrotic programmed cell death characterized by phosphorylation signal pathway mediated by receptor-interacting serine/threonine-protein kinase 3 (RIPK3), which activates mixed lineage kinase domain-like protein (MLKL) (Declercq et al., 2009; Marshall and Baines, 2014). Previous studies have shown that RIPK1, which is an independent necroptosis factor, plays a significant role in modulating autophagic signaling (Lu and Walsh, 2012), suggesting it promotes autophagy. Morphologically, necroptosis is characterized by necrotic cell death, such as lysosome membrane degradation, vacuolation of cytoplasm, disintegration of plasma membrane, and explosion of cells. Although traditional chemotherapeutic drugs have shown efficacy in inhibiting tumor growth by inducing apoptosis of tumor cells (Han et al., 2007), these cells often develop certain drug resistance due to the imbalance of their own apoptosis mechanism. Previous studies have suggested that apoptosis-resistant tumor cells may be sensitive to necroptosis pathway (Su et al., 2016; He et al., 2017), indicating that the study of necroptosis and its regulatory mechanism can be a target for development of treatment for cancer.

To date, however, little is known about the role of necroptosis in the pathogenesis and progression of PCa. Availability of large-scale databases has provided us with effective measures for exploration of gene signatures and clinical phenotype, thereby enhancing our understanding of the relationship between necroptosis and PCa. In the present study, we first downloaded and combined datasets from TCGA and GTEx databases, then compared mRNA expression levels between tumor (TCGA database) and normal pancreatic tissues (GTEx database), because the number of normal pancreatic tissues in the TCGA database was limited. Finally, we obtained 132 differentially expressed NRGs. GO terms and KEGG pathway analysis of these differentially expressed NRGs revealed that most of them were involved in regulating necroptosis. In order to analysis PCa prognosis-related genes from the perspective of necroptosis, we screened and identified five prognostic NRGs using univariate and multivariate Cox regression analysis. The identified five prognosis-related NRGs could be used to establish a prognostic signature for successfully stratifying PCa patients into two groups. Results showed patients in the high-risk group had significantly lower OS than their low-risk counterparts. Results from multivariate Cox regression analysis confirmed the independent prognostic value of the established necroptosis-related signature, with its risk score correlated with survival outcomes, age, and lymph node metastasis. Besides, stratification analysis based on age, gender, and tumor stage in patients in high-risk group had significantly shorter OS than those in the low-risk group, indicating that the necroptosis-related signature had excellent prognostic value in predicting pathogenesis and progression of PCa patients.

Our results further suggested that all genes in the signature were closely related to prognosis of PCa. One such gene was GULD1, which is one of the key genes regulating glutamine-dependent metabolism and has been proven to support the growth of pancreatic cancer (Son et al., 2013). Previous studies have also shown that the glutamine-dependent metabolism is significantly associated with shorter OS of PCa patients (Yu et al., 2015). Another gene, PYGL, a glycolysis-related gene, is highly expressed in PCa patients compared with normal tissues and confirmed using immunohistochemistry, and could predict the prognosis of patients with PCa (Nie et al., 2021). However, SPATA2, H2AC8, and TNFSF10 had not been previously studied regarding prognostic value of PCa patients. GSEA analysis, targeting differentially expressed genes in the GEO dataset, verified several necroptosis-related pathways. Particularly, focal adhesion kinase (FAK) plays a key role in regulating cell migration, invasion, anchorage-independent growth during cancer cell metastasis (Sulzmaier et al., 2014). Previous studies have also shown that FAK inhibition increases immune surveillance by overcoming the fibrotic and immunosuppressive pancreatic tumor microenvironment, which renders tumors responsive to immunotherapy (Jiang et al., 2016). Additional research evidences have also shown that PI3K/AKT signaling pathway is associated with increased cancer cell proliferation and survival (Song et al., 2019), thus targeting PI3K/AKT signaling pathways could provide an important approach for PCa prevention and therapy (Xu et al., 2019). In addition, studies have shown that the PI3K/AKT signaling pathway modulates induction of apoptosis and necroptosis (Wang et al., 2020). In the present study, Kaplan-Meier curves of OS and ROC analysis in the GSE71729 validated that necroptosis-related signature could be an independent prognostic indicator.

In conclusion, we had established a necroptosis-related gene signature that can be used to predict prognosis in patients with PCa. We had successfully validated this model using an independent GEO cohort. Taken together, these finding provide new insights into the necroptosis status in PCa and even new targets for PCa. Further explorations are needed to validate this signature and ascertain its application in clinical treatments.

## Data Availability

The original contributions presented in the study are included in the article/Supplementary Material, further inquiries can be directed to the corresponding author.
