# Supplementary material for: An Efficient Signature Based on Necroptosis-Related Genes for Prognosis of Patients With Pancreatic Cancer
Source: Front Genet. 2022 Mar 28;13:848747. doi: 10.3389/fgene.2022.848747 (PMC8995900; doi:10.3389/fgene.2022.848747)
Supplement: Supplementary file 1 [file Table1.docx]

**Supplement Table 1** Human necroptosis-related gene set in KEGG

| **Entrez ID** | **Gene Symbol** | **Gene name** |
| --- | --- | --- |
| 7124 | TNF | tumor necrosis factor |
| 7132 | TNFRSF1A | TNF receptor superfamily member 1A |
| 8717 | TRADD | TNFRSF1A associated via death domain |
| 7186 | TRAF2 | TNF receptor associated factor 2 |
| 7188 | TRAF5 | TNF receptor associated factor 5 |
| 8737 | RIPK1 | receptor interacting serine/threonine kinase 1 |
| 329 | BIRC2 | baculoviral IAP repeat containing 2 |
| 330 | BIRC3 | baculoviral IAP repeat containing 3 |
| 331 | XIAP | X-linked inhibitor of apoptosis |
| 10616 | RBCK1 | RANBP2-type and C3HC4-type zinc finger containing 1 |
| 55072 | RNF31 | ring finger protein 31 |
| 81858 | SHARPIN | SHANK associated RH domain interactor |
| 124044 | SPATA2L | spermatogenesis associated 2 like |
| 9825 | SPATA2 | spermatogenesis associated 2 |
| 1540 | CYLD | CYLD lysine 63 deubiquitinase |
| 8772 | FADD | Fas associated via death domain |
| 841 | CASP8 | caspase 8 |
| 8837 | CFLAR | CASP8 and FADD like apoptosis regulator |
| 11035 | RIPK3 | receptor interacting serine/threonine kinase 3 |
| 1536 | CYBB | cytochrome b-245 beta chain |
| 815 | CAMK2A | calcium/calmodulin dependent protein kinase II alpha |
| 817 | CAMK2D | calcium/calmodulin dependent protein kinase II delta |
| 816 | CAMK2B | calcium/calmodulin dependent protein kinase II beta |
| 818 | CAMK2G | calcium/calmodulin dependent protein kinase II gamma |
| 291 | SLC25A4 | solute carrier family 25 member 4 |
| 292 | SLC25A5 | solute carrier family 25 member 5 |
| 293 | SLC25A6 | solute carrier family 25 member 6 |
| 83447 | SLC25A31 | solute carrier family 25 member 31 |
| 5481 | PPID | peptidylprolyl isomerase D |
| 7416 | VDAC1 | voltage dependent anion channel 1 |
| 7417 | VDAC2 | voltage dependent anion channel 2 |
| 7419 | VDAC3 | voltage dependent anion channel 3 |
| 2747 | GLUD2 | glutamate dehydrogenase 2 |
| 2746 | GLUD1 | glutamate dehydrogenase 1 |
| 2752 | GLUL | glutamate-ammonia ligase |
| 5836 | PYGL | glycogen phosphorylase L |
| 5837 | PYGM | glycogen phosphorylase, muscle associated |
| 5834 | PYGB | glycogen phosphorylase B |
| 5599 | MAPK8 | mitogen-activated protein kinase 8 |
| 5602 | MAPK10 | mitogen-activated protein kinase 10 |
| 5601 | MAPK9 | mitogen-activated protein kinase 9 |
| 2495 | FTH1 | ferritin heavy chain 1 |
| 2512 | FTL | ferritin light chain |
| 123745 | PLA2G4E | phospholipase A2 group IVE |
| 5321 | PLA2G4A | phospholipase A2 group IVA |
| 8681 | JMJD7-PLA2G4B | JMJD7-PLA2G4B readthrough |
| 100137049 | PLA2G4B | phospholipase A2 group IVB |
| 8605 | PLA2G4C | phospholipase A2 group IVC |
| 283748 | PLA2G4D | phospholipase A2 group IVD |
| 255189 | PLA2G4F | phospholipase A2 group IVF |
| 246 | ALOX15 | arachidonate 15-lipoxygenase |
| 823 | CAPN1 | calpain 1 |
| 824 | CAPN2 | calpain 2 |
| 6609 | SMPD1 | sphingomyelin phosphodiesterase 1 |
| 197259 | MLKL | mixed lineage kinase domain like pseudokinase |
| 192111 | PGAM5 | PGAM family member 5, mitochondrial serine/threonine protein phosphatase |
| 10059 | DNM1L | dynamin 1 like |
| 114548 | NLRP3 | NLR family pyrin domain containing 3 |
| 29108 | PYCARD | PYD and CARD domain containing |
| 834 | CASP1 | caspase 1 |
| 3553 | IL1B | interleukin 1 beta |
| 27243 | CHMP2A | charged multivesicular body protein 2A |
| 25978 | CHMP2B | charged multivesicular body protein 2B |
| 51652 | CHMP3 | charged multivesicular body protein 3 |
| 100526767 | RNF103-CHMP3 | RNF103-CHMP3 readthrough |
| 128866 | CHMP4B | charged multivesicular body protein 4B |
| 29082 | CHMP4A | charged multivesicular body protein 4A |
| 92421 | CHMP4C | charged multivesicular body protein 4C |
| 79643 | CHMP6 | charged multivesicular body protein 6 |
| 9525 | VPS4B | vacuolar protein sorting 4 homolog B |
| 27183 | VPS4A | vacuolar protein sorting 4 homolog A |
| 57132 | CHMP1B | charged multivesicular body protein 1B |
| 5119 | CHMP1A | charged multivesicular body protein 1A |
| 51510 | CHMP5 | charged multivesicular body protein 5 |
| 91782 | CHMP7 | charged multivesicular body protein 7 |
| 54822 | TRPM7 | transient receptor potential cation channel subfamily M member 7 |
| 3552 | IL1A | interleukin 1 alpha |
| 90865 | IL33 | interleukin 33 |
| 3146 | HMGB1 | high mobility group box 1 |
| 8743 | TNFSF10 | TNF superfamily member 10 |
| 8797 | TNFRSF10A | TNF receptor superfamily member 10a |
| 8795 | TNFRSF10B | TNF receptor superfamily member 10b |
| 356 | FASLG | Fas ligand |
| 355 | FAS | Fas cell surface death receptor |
| 11124 | FAF1 | Fas associated factor 1 |
| 3439 | IFNA1 | interferon alpha 1 |
| 3440 | IFNA2 | interferon alpha 2 |
| 3441 | IFNA4 | interferon alpha 4 |
| 3442 | IFNA5 | interferon alpha 5 |
| 3443 | IFNA6 | interferon alpha 6 |
| 3444 | IFNA7 | interferon alpha 7 |
| 3445 | IFNA8 | interferon alpha 8 |
| 3446 | IFNA10 | interferon alpha 10 |
| 3447 | IFNA13 | interferon alpha 13 |
| 3448 | IFNA14 | interferon alpha 14 |
| 3449 | IFNA16 | interferon alpha 16 |
| 3451 | IFNA17 | interferon alpha 17 |
| 3452 | IFNA21 | interferon alpha 21 |
| 3456 | IFNB1 | interferon beta 1 |
| 3458 | IFNG | interferon gamma |
| 3454 | IFNAR1 | interferon alpha and beta receptor subunit 1 |
| 3455 | IFNAR2 | interferon alpha and beta receptor subunit 2 |
| 3459 | IFNGR1 | interferon gamma receptor 1 |
| 3460 | IFNGR2 | interferon gamma receptor 2 |
| 3716 | JAK1 | Janus kinase 1 |
| 3717 | JAK2 | Janus kinase 2 |
| 3718 | JAK3 | Janus kinase 3 |
| 7297 | TYK2 | tyrosine kinase 2 |
| 6772 | STAT1 | signal transducer and activator of transcription 1 |
| 6773 | STAT2 | signal transducer and activator of transcription 2 |
| 6774 | STAT3 | signal transducer and activator of transcription 3 |
| 6775 | STAT4 | signal transducer and activator of transcription 4 |
| 6776 | STAT5A | signal transducer and activator of transcription 5A |
| 6777 | STAT5B | signal transducer and activator of transcription 5B |
| 6778 | STAT6 | signal transducer and activator of transcription 6 |
| 10379 | IRF9 | interferon regulatory factor 9 |
| 5610 | EIF2AK2 | eukaryotic translation initiation factor 2 alpha kinase 2 |
| 7099 | TLR4 | toll like receptor 4 |
| 353376 | TICAM2 | toll like receptor adaptor molecule 2 |
| 148022 | TICAM1 | toll like receptor adaptor molecule 1 |
| 7098 | TLR3 | toll like receptor 3 |
| 81030 | ZBP1 | Z-DNA binding protein 1 |
| 27005 | USP21 | ubiquitin specific peptidase 21 |
| 8878 | SQSTM1 | sequestosome 1 |
| 3320 | HSP90AA1 | heat shock protein 90 alpha family class A member 1 |
| 3326 | HSP90AB1 | heat shock protein 90 alpha family class B member 1 |
| 7128 | TNFAIP3 | TNF alpha induced protein 3 |
| 142 | PARP1 | poly(ADP-ribose) polymerase 1 |
| 637 | BID | BH3 interacting domain death agonist |
| 581 | BAX | BCL2 associated X, apoptosis regulator |
| 9131 | AIFM1 | apoptosis inducing factor mitochondria associated 1 |
| 3014 | H2AX | H2A.X variant histone |
| 8338 | H2AC20 | H2A clustered histone 20 |
| 85235 | H2AC12 | H2A clustered histone 12 |
| 221613 | H2AC1 | H2A clustered histone 1 |
| 92815 | H2AW | H2A.W histone |
| 83740 | H2AB3 | H2A.B variant histone 3 |
| 3012 | H2AC8 | H2A clustered histone 8 |
| 8335 | H2AC4 | H2A clustered histone 4 |
| 55506 | MACROH2A2 | macroH2A.2 histone |
| 9555 | MACROH2A1 | macroH2A.1 histone |
| 723790 | H2AC19 | H2A clustered histone 19 |
| 55766 | H2AJ | H2A.J histone |
| 474382 | H2AB1 | H2A.B variant histone 1 |
| 8336 | H2AC17 | H2A clustered histone 17 |
| 8337 | H2AC18 | H2A clustered histone 18 |
| 8969 | H2AC11 | H2A clustered histone 11 |
| 317772 | H2AC21 | H2A clustered histone 21 |
| 94239 | H2AZ2 | H2A.Z variant histone 2 |
| 3013 | H2AC7 | H2A clustered histone 7 |
| 3015 | H2AZ1 | H2A.Z variant histone 1 |
| 8330 | H2AC15 | H2A clustered histone 15 |
| 8334 | H2AC6 | H2A clustered histone 6 |
| 8329 | H2AC13 | H2A clustered histone 13 |
| 8331 | H2AC14 | H2A clustered histone 14 |
| 8332 | H2AC16 | H2A clustered histone 16 |
| 474381 | H2AB2 | H2A.B variant histone 2 |
| 5478 | PPIA | peptidylprolyl isomerase A |
| 596 | BCL2 | BCL2 apoptosis regulator |
