# Supplementary material for: An Efficient Signature Based on Necroptosis-Related Genes for Prognosis of Patients With Pancreatic Cancer
Source: Front Genet. 2022 Mar 28;13:848747. doi: 10.3389/fgene.2022.848747 (PMC8995900; doi:10.3389/fgene.2022.848747)
Supplement: Supplementary file 2 [file Table2.docx]

**Supplement Table 2** Differentially expressed NRGs between tumor and normal tissues

| **Gene Symbol** | **logFC** | **P.Value** | **adj.P.Val** | **Type** |
| --- | --- | --- | --- | --- |
| IFNA1 | 9.787051 | 2.58E-271 | 3.56E-269 | Upregulated |
| H2AC14 | 9.600439 | 1.80E-209 | 1.24E-207 | Upregulated |
| H2AC16 | 9.563059 | 4.57E-202 | 2.10E-200 | Upregulated |
| H2AC13 | 9.896051 | 1.00E-168 | 3.45E-167 | Upregulated |
| IFNG | 8.945226 | 9.02E-166 | 2.49E-164 | Upregulated |
| H2AC4 | 9.105529 | 1.11E-157 | 2.55E-156 | Upregulated |
| GLUD1 | 36.30484 | 1.16E-138 | 2.28E-137 | Upregulated |
| H2AC21 | 8.819099 | 4.10E-137 | 7.08E-136 | Upregulated |
| CHMP4B | 135.9064 | 4.83E-135 | 7.40E-134 | Upregulated |
| PLA2G4E | 6.394645 | 1.94E-134 | 2.68E-133 | Upregulated |
| PLA2G4D | 7.063288 | 3.86E-134 | 4.84E-133 | Upregulated |
| HMGB1 | 19.33202 | 1.22E-133 | 1.41E-132 | Upregulated |
| IFNAR1 | 10.78808 | 3.68E-133 | 3.90E-132 | Upregulated |
| SLC25A6 | 204.7948 | 4.20E-126 | 4.14E-125 | Upregulated |
| CHMP1A | 36.98806 | 1.03E-125 | 9.47E-125 | Upregulated |
| HSP90AB1 | 343.3038 | 5.00E-124 | 4.31E-123 | Upregulated |
| CAMK2A | 4.719191 | 3.13E-122 | 2.54E-121 | Upregulated |
| H2AZ2 | 18.48322 | 2.28E-121 | 1.75E-120 | Upregulated |
| STAT3 | 24.23323 | 2.62E-121 | 1.90E-120 | Upregulated |
| VDAC3 | 17.20071 | 2.96E-120 | 2.04E-119 | Upregulated |
| PPIA | 59.63351 | 6.54E-120 | 4.30E-119 | Upregulated |
| H2AC17 | 8.650317 | 9.75E-120 | 6.11E-119 | Upregulated |
| SLC25A5 | 153.22 | 1.34E-118 | 8.05E-118 | Upregulated |
| H2AC12 | 8.376676 | 8.58E-114 | 4.93E-113 | Upregulated |
| JAK1 | 30.4517 | 1.57E-111 | 8.69E-111 | Upregulated |
| RIPK1 | 7.887833 | 5.45E-111 | 2.89E-110 | Upregulated |
| H2AZ1 | 47.82469 | 8.46E-110 | 4.33E-109 | Upregulated |
| ALOX15 | 7.347945 | 5.59E-109 | 2.75E-108 | Upregulated |
| H2AC11 | 6.045849 | 1.97E-108 | 9.37E-108 | Upregulated |
| CHMP5 | 27.97781 | 1.46E-107 | 6.73E-107 | Upregulated |
| CHMP1B | 21.48212 | 1.33E-106 | 5.91E-106 | Upregulated |
| VDAC1 | 71.92781 | 1.85E-106 | 7.97E-106 | Upregulated |
| SQSTM1 | 34.15137 | 5.12E-106 | 2.14E-105 | Upregulated |
| FTH1 | 206.9397 | 5.72E-104 | 2.32E-103 | Upregulated |
| TNFRSF1A | 41.79638 | 8.60E-104 | 3.39E-103 | Upregulated |
| CHMP2A | 56.09522 | 6.21E-103 | 2.38E-102 | Upregulated |
| PARP1 | 13.35268 | 8.72E-103 | 3.25E-102 | Upregulated |
| CHMP2B | 17.6791 | 1.32E-98 | 4.79E-98 | Upregulated |
| CAPN2 | 45.86871 | 1.71E-98 | 6.04E-98 | Upregulated |
| BIRC2 | 10.65754 | 2.24E-98 | 7.72E-98 | Upregulated |
| RNF103-CHMP3 | 7.726808 | 2.42E-98 | 8.14E-98 | Upregulated |
| VPS4B | 9.158448 | 9.97E-98 | 3.27E-97 | Upregulated |
| FASLG | 6.071426 | 1.26E-97 | 4.03E-97 | Upregulated |
| CAPN1 | 34.19076 | 1.38E-97 | 4.33E-97 | Upregulated |
| IFNGR2 | 44.57886 | 4.26E-97 | 1.31E-96 | Upregulated |
| IFNGR1 | 36.96992 | 4.42E-97 | 1.33E-96 | Upregulated |
| IL1A | 8.406668 | 3.05E-96 | 8.96E-96 | Upregulated |
| VPS4A | 6.59681 | 1.71E-95 | 4.91E-95 | Upregulated |
| STAT6 | 25.20341 | 7.41E-95 | 2.09E-94 | Upregulated |
| CHMP3 | 9.412268 | 3.53E-94 | 9.73E-94 | Upregulated |
| AIFM1 | 7.660065 | 4.91E-94 | 1.33E-93 | Upregulated |
| MACROH2A1 | 7.362301 | 1.16E-92 | 3.07E-92 | Upregulated |
| PGAM5 | 6.724947 | 5.82E-90 | 1.51E-89 | Upregulated |
| XIAP | 5.883875 | 6.89E-89 | 1.76E-88 | Upregulated |
| CHMP6 | 7.72916 | 2.65E-88 | 6.66E-88 | Upregulated |
| STAT2 | 13.87235 | 4.88E-87 | 1.20E-86 | Upregulated |
| BAX | 22.29622 | 3.80E-86 | 9.20E-86 | Upregulated |
| EIF2AK2 | 5.979873 | 1.17E-85 | 2.78E-85 | Upregulated |
| SHARPIN | 19.98139 | 3.91E-84 | 9.14E-84 | Upregulated |
| TRAF2 | 7.357575 | 6.65E-83 | 1.53E-82 | Upregulated |
| GLUD2 | 2.142469 | 8.75E-82 | 1.98E-81 | Upregulated |
| RBCK1 | 21.21139 | 3.24E-81 | 7.21E-81 | Upregulated |
| PLA2G4F | 3.210214 | 1.04E-80 | 2.28E-80 | Upregulated |
| FTL | 3220.133 | 2.43E-80 | 5.23E-80 | Upregulated |
| ZBP1 | 4.628348 | 3.44E-80 | 7.31E-80 | Upregulated |
| NLRP3 | 3.380075 | 5.64E-80 | 1.18E-79 | Upregulated |
| FADD | 4.877835 | 1.01E-78 | 2.07E-78 | Upregulated |
| DNM1L | 6.021605 | 1.34E-78 | 2.73E-78 | Upregulated |
| CHMP7 | 8.411095 | 4.40E-78 | 8.79E-78 | Upregulated |
| BID | 5.986793 | 8.48E-77 | 1.67E-76 | Upregulated |
| VDAC2 | 15.76619 | 1.36E-76 | 2.64E-76 | Upregulated |
| TNF | 5.090342 | 2.47E-76 | 4.73E-76 | Upregulated |
| MACROH2A2 | 10.84889 | 3.87E-76 | 7.31E-76 | Upregulated |
| GLUL | 56.28559 | 7.98E-75 | 1.49E-74 | Upregulated |
| H2AC7 | 7.749869 | 2.53E-74 | 4.66E-74 | Upregulated |
| STAT5B | 8.628037 | 3.03E-74 | 5.50E-74 | Upregulated |
| TICAM1 | 16.1153 | 3.88E-74 | 6.96E-74 | Upregulated |
| CAMK2D | 6.538567 | 2.81E-73 | 4.98E-73 | Upregulated |
| STAT5A | 6.534669 | 3.21E-73 | 5.61E-73 | Upregulated |
| SMPD1 | 15.95008 | 7.45E-70 | 1.29E-69 | Upregulated |
| HSP90AA1 | 190.8973 | 1.80E-69 | 3.07E-69 | Upregulated |
| TNFRSF10A | 7.124905 | 1.62E-68 | 2.73E-68 | Upregulated |
| SPATA2 | 3.608562 | 3.79E-67 | 6.22E-67 | Upregulated |
| TRADD | 12.52979 | 5.20E-67 | 8.44E-67 | Upregulated |
| H2AX | 17.88019 | 1.06E-64 | 1.70E-64 | Upregulated |
| TLR4 | 4.266073 | 1.93E-63 | 3.07E-63 | Upregulated |
| PPID | 7.401497 | 1.97E-63 | 3.10E-63 | Upregulated |
| IFNAR2 | 5.767959 | 2.37E-60 | 3.63E-60 | Upregulated |
| TYK2 | 7.244209 | 1.68E-59 | 2.55E-59 | Upregulated |
| FAS | 4.928758 | 4.14E-59 | 6.22E-59 | Upregulated |
| CHMP4C | 11.45905 | 1.80E-58 | 2.67E-58 | Upregulated |
| PYCARD | 16.61094 | 2.18E-55 | 3.20E-55 | Upregulated |
| JAK2 | 3.442289 | 4.96E-54 | 7.21E-54 | Upregulated |
| CASP1 | 5.183847 | 4.40E-53 | 6.33E-53 | Upregulated |
| H2AC6 | 25.51023 | 7.33E-52 | 1.04E-51 | Upregulated |
| STAT1 | 32.75097 | 9.97E-52 | 1.40E-51 | Upregulated |
| USP21 | 3.354531 | 1.12E-51 | 1.56E-51 | Upregulated |
| TNFRSF10B | 15.56001 | 3.02E-51 | 4.17E-51 | Upregulated |
| PYGB | 71.8726 | 1.04E-50 | 1.42E-50 | Upregulated |
| H2AJ | 14.12 | 1.85E-50 | 2.51E-50 | Upregulated |
| IL1B | 3.215178 | 1.76E-45 | 2.35E-45 | Upregulated |
| SPATA2L | 4.680377 | 1.88E-44 | 2.49E-44 | Upregulated |
| TLR3 | 2.446086 | 1.92E-43 | 2.53E-43 | Upregulated |
| BCL2 | 2.75488 | 3.93E-43 | 5.12E-43 | Upregulated |
| H2AC8 | 6.500933 | 3.58E-42 | 4.62E-42 | Upregulated |
| TNFAIP3 | 10.63301 | 9.57E-42 | 1.22E-41 | Upregulated |
| PLA2G4A | 6.031547 | 1.82E-40 | 2.29E-40 | Upregulated |
| BIRC3 | 16.98885 | 1.06E-39 | 1.32E-39 | Upregulated |
| CASP8 | 2.580014 | 1.49E-39 | 1.84E-39 | Upregulated |
| PYGL | 7.048474 | 1.17E-38 | 1.43E-38 | Upregulated |
| CYLD | 2.482435 | 2.24E-37 | 2.71E-37 | Upregulated |
| CAMK2G | 4.562208 | 2.60E-37 | 3.12E-37 | Upregulated |
| MLKL | 2.128489 | 1.87E-36 | 2.22E-36 | Upregulated |
| CYBB | 13.24115 | 5.61E-35 | 6.62E-35 | Upregulated |
| CFLAR | 3.07524 | 1.92E-34 | 2.24E-34 | Upregulated |
| RIPK3 | 4.136691 | 2.20E-34 | 2.55E-34 | Upregulated |
| JAK3 | 4.493299 | 8.50E-32 | 9.78E-32 | Upregulated |
| IL33 | 5.975542 | 4.71E-27 | 5.32E-27 | Upregulated |
| TNFSF10 | 24.85981 | 7.29E-27 | 8.18E-27 | Upregulated |
| STAT4 | 1.645711 | 1.84E-24 | 2.05E-24 | Upregulated |
| MAPK9 | 1.576711 | 6.19E-24 | 6.84E-24 | Upregulated |
| FAF1 | 1.731068 | 2.36E-23 | 2.59E-23 | Upregulated |
| H2AC20 | 1.836113 | 5.38E-23 | 5.85E-23 | Upregulated |
| PYGM | 1.030256 | 1.63E-18 | 1.76E-18 | Upregulated |
| TRPM7 | 1.200321 | 1.10E-16 | 1.18E-16 | Upregulated |
| SLC25A4 | 3.381418 | 1.87E-15 | 1.99E-15 | Upregulated |
| H2AW | 10.21762 | 2.38E-15 | 2.51E-15 | Upregulated |
| CAMK2B | 1.78141 | 1.85E-05 | 1.93E-05 | Upregulated |
| CHMP4A | -3.03903 | 8.61E-68 | 1.43E-67 | Downregulated |
| PLA2G4B | -2.39451 | 4.42E-63 | 6.86E-63 | Downregulated |
| JMJD7-PLA2G4B | -1.71936 | 7.42E-41 | 9.40E-41 | Downregulated |
| IRF9 | -2.15412 | 1.43E-30 | 1.63E-30 | Downregulated |
