# Supplementary material for: An Efficient Signature Based on Necroptosis-Related Genes for Prognosis of Patients With Pancreatic Cancer
Source: Front Genet. 2022 Mar 28;13:848747. doi: 10.3389/fgene.2022.848747 (PMC8995900; doi:10.3389/fgene.2022.848747)
Supplement: Supplementary file 3 [file Table3.docx]

**Supplement Table 3** The five NRGs for the construction of the prognostic model

| Gene symbol | coefficient | Hazard ration (HR) | HR.95L | HR.95H | p-value |
| --- | --- | --- | --- | --- | --- |
| GLUD1 | 0.024261842 | 1.024558555 | 1.000567613 | 1.049124736 | 0.04476002 |
| SPATA2 | -0.22825263 | 0.79592316 | 0.664603078 | 0.953191006 | 0.01309915 |
| H2AC8 | -0.133607826 | 0.874933123 | 0.776169117 | 0.986264402 | 0.02879487 |
| PYGL | 0.053314383 | 1.054761192 | 1.014282348 | 1.096855501 | 0.007579941 |
| TNFSF10 | 0.010418899 | 1.010473365 | 1.003002688 | 1.017999685 | 0.005925998 |
